# Facile and Robust Solvothermal Synthesis of Nanocrystalline CuInS_2_ Thin Films

**DOI:** 10.3390/nano8060405

**Published:** 2018-06-05

**Authors:** Anna Frank, Jan Grunwald, Benjamin Breitbach, Christina Scheu

**Affiliations:** 1 Max-Planck-Institut für Eisenforschung GmbH, Max-Planck-Straße 1, 40237 Düsseldorf, Germany; frank@mpie.de (A.F.); breitbach@mpie.de (B.B.); 2 Ludwig-Maximilians-Universität, Butenandtstraße 5-11, 81377 Munich, Germany; jan_grunwald92@gmx.de; 3 Materials Analytics, RWTH Aachen University, Kopernikusstraße 10, 52074 Aachen, Germany

**Keywords:** solvothermal synthesis, CuInS_2_, TEM

## Abstract

This work demonstrates that the solvothermal synthesis of nanocrystalline CuInS_2_ thin films using the amino acid l-cysteine as sulfur source is facile and robust against variation of reaction time and temperature. Synthesis was carried out in a reaction time range of 3–48 h (at 150 °C) and a reaction temperature range of 100–190 °C (for 18 h). It was found that at least a time of 6 h and a temperature of 140 °C is needed to produce pure nanocrystalline CuInS_2_ thin films as proven by X-ray and electron diffraction, high-resolution transmission electron microscopy, and energy-dispersive X-ray spectroscopy. Using UV-vis spectroscopy, a good absorption behavior as well as direct band gaps between 1.46 and 1.55 eV have been determined for all grown films. Only for a reaction time of 3 h and temperatures below 140 °C CuInS_2_ is not formed. This is attributed to the formation of metal ion complexes with l-cysteine and the overall slow assembly of CuInS_2_. This study reveals that the reaction parameters can be chosen relatively free; the reaction is completely nontoxic and precursors and solvents are rather cheap, which makes this synthesis route interesting for industrial up scaling.

## 1. Introduction

Due to the growing energy needs of our society, the scarcity of fossil fuels, and threatening greenhouse effect, research on materials that offer appropriate functionalities to overcome these problems is desperately needed; this is therefore a very active research field. Possible applications involve the generation of electricity via solar energy, the production of alternative fuels like hydrogen, and the decomposition of contaminants; but involves also research on how to store the produced energy [1,2,3,4,5,6,7]. For example, the photosynthesis of plants is mimicked to split water by light [8,9] or to convert CO_2_ into less-harmful compounds [10]. To keep the costs economic, the synthesis of green energy materials should also be a green synthesis. Such a synthesis should be feasible without the need for expensive precursors, high pressures, or temperatures (i.e., high energy input), using a route that tolerates deviations in temperature and time, ideally accomplished in only one synthesis step, and should also avoid toxic chemicals during preparation, [11,12]. The use of biomolecules as precursors in chemical reactions and the formation of nanomaterials for diverse applications has been actively investigated in recent times [4,6,12,13,14,15,16,17].

Copper indium disulfide, CuInS_2_, is a material suitable for diverse solar-driven applications [18,19,20]. It offers a band gap of 1.5 eV for the bulk, a high-absorption coefficient (α = 10^5^ cm^−1^) [21] and can be used to convert sunlight into electricity or as a photocatalyst. CuInS_2_ can be fabricated with various techniques which, in most cases, require high temperatures, high pressure, and clean precursor metals, but also with wet-chemical approaches [22,23,24,25]. We chose the solvothermal route to prepare CuInS_2_, as it can be considered a green synthesis route—it only uses simple solvents and metal salts, while achieving a wide variety of nanostructures at low-reaction temperatures [26,27]. Furthermore, the solvothermal growth allows for the direct, one-pot CuInS_2_ deposition on a suitable substrate—growing (thin) films in-situ without the need to deposit synthesized material afterwards [28].

Peng et al. [28] developed a solvothermal synthesis strategy for growing CuInS_2_ films directly on fluorine-doped tin oxide (FTO) using simple salts as precursors. This synthesis strategy has been used and slightly modified in our group to prepare CuInS_2_ thin films as well as microspheres [29,30]. However, this synthesis route involves the carcinogenic substance thioacetamide as sulfur source [31]. Therefore, we recently changed the sulfur source to the natural amino acid l-cysteine to achieve a complete non-toxic, green synthesis pathway towards CuInS_2_ films on FTO substrates [32]. There, we varied the concentration and ratio of the used precursor salts to investigate the influence on sample morphology and properties, while keeping the reaction conditions constant (150 °C, 18 h). In short, at high sulfur ratios, an additional nanoflake layer of In_2_S_3_ on top of a compact CuInS_2_ film was observed [32]. On reviewing literature, it becomes clear that it is possible to synthesize CuInS_2_ thin films in a wide variety of reaction conditions and with many possible precursor salts and solvents; e.g., Peng et al. [28] used CuSO_4_, InCl_3_ and thioacetamide in ethanol at 160 °C for 12 h to produce pure CuInS_2_. Wochnik et al. [29] were also able to synthesize pure tetragonal CuInS_2_ films but at 150 °C for 24 h using the same precursors and solvent. Furthermore, it is possible to produce pure and stoichiometric CuInS_2_ thin films with CuCl_2_, In(NO_3_)_3_, thiourea, CTAB and oxalic acid in ethanol at 200 °C for 24 h as demonstrated by Xia et al. [33] The synthesis from Cu_2_O, In(OH)_3_, thioacetic acid and ammonia in ethanol at 150 °C for 6 h is also possible, as shown by Liu et al. [34] Solvothermal synthesis of nanostructured CuInS_2_ using l-cysteine as a sulfur source has already been published in literature [35,36]. Liu et al. [35] reported about the formation of CuInS_2_ using CuCl_2_, InCl_3_ and l-cystine in 1:1 ethylene diamine: water. They kept the autoclave at 200 °C for 12 h, resulting in microspheres and nanoparticles in the tetragonal Chalcopyrite structure without any visible impurities or side products. The composition of their CuInS_2_ samples was also in a stoichiometric range. Wen et al. [36] synthesized CuInS_2_ microspheres out of CuCl_2_, InCl_2_ and l-cysteine in *N*,*N*-dimethylformamide (DMF) as a solvent, also at 200 °C for 12 h. Their product displayed the tetragonal Chalcopyrite modification as well.

The results in literature indicate that pure, crystalline CuInS_2_ nanostructures can be fabricated within a relative large reaction window; however, systematic studies, where the reaction temperature and time are varied in a broad range while keeping all other parameters constant, are rare. Additionally, many of the already existing synthesis routes involve toxic substances as raw materials.

In the present work, we fill that gap and focus on the influence of the reaction temperature and time on the l-cysteine-assisted solvothermal growth of nanocrystalline CuInS_2_ films. We show that the solvothermal synthesis of CuInS_2_ using l-cysteine as sulfur source is not only non-toxic but also extremely robust over a large temperature range from 140 °C to 190 °C as well as less critical on large time variations from 6 to 48 h. Thus, this synthesis pathway is very interesting for possible industrial utilization.

## 2. Materials and Methods

### 2.1. Synthesis of CuInS_2_ Films

Chemicals were used as-purchased from Sigma-Aldrich (Sigma-Aldrich Chemie Gmbh, Munich, Germany) without further purification. The FTO glass substrates (Sigma-Aldrich) were cut into pieces of 15 mm × 20 mm × 2 mm, cleaned ultrasonically in dilute nitric acid, double-distilled water, acetone and ethanol for 5 min each prior to synthesis. The films were grown with our recently reported synthesis strategy using l-cysteine as sulfur source [32], which is based on the method published by Peng et al. [28] and our group [29] where thioacetamide was used.

The procedure is as follows: CuSO_4_∙5H_2_O (0.2 mol, 0.050 g) and InCl_3_ (0.2 mol, 0.044 g) were weighed out directly into a Teflon liner (20 mL capacity) and dissolved in 10 mL ethanol. The mixture was stirred for 10 min after which l-cysteine (0.5 mol, 0.061 g) was added. After stirring for another 5 min, a piece of FTO was placed inside the Teflon liner, conducting side facing down, the stainless-steel autoclave was sealed and put into an electric oven. There it was kept for 3, 6, 9, 12, 15, 18, 21, 24 and 48 h at a temperature of 150 °C and for 18 h at temperatures of 100, 120, 140, 150, 160, 180 and 190 °C. That means that for a variation of the reaction time, the reaction temperature was kept constant at 150 °C and for a variation of the reaction temperature the reaction time was always 18 h. The ratio between the precursors was kept at Cu:In:S 1:1:2.5. The film grown at this concentration/ratio and at 150 °C for 18 h has been published before [32] and will be referred to as film_S (standard reaction conditions). The other films are named according to their variation in time or temperature as film_time or film_temperature.

### 2.2. Characterization

To investigate the crystal structure of the synthesized CuInS_2_ films on a global scale, X-ray diffraction (XRD) was used. To minimize the contribution from the FTO substrate, the measurements were performed under grazing incidence geometry with an incident angle of α = 2° in a Seifert THETA/2THETA X-ray diffractometer. The diffractometer was equipped with a Co source (λKα = 1.79 Å), polycapillary beam optics and an energy dispersive point detector. The 2θ values ranging from 10° to 140° were measured with a step size of 0.05°/s and a count time of 30 s/step. The X-ray generator was operated at 40 kV and 30 mA. Literature data were used to identify the obtained phases. To calculate the average crystallite size, the Scherrer equation [37] was applied, fitting the most intense CuInS_2_ peaks (112) and (204) with a Gaussian function.

The morphology of the CuInS_2_ films was evaluated using scanning electron microscopy (SEM). For this purpose, a ZEISS Merlin, operated at 5.0 kV and a probe current of 2.0 nA, was used. Imaging was performed using the attached InLens^®^ ZEISS standard detector. To analyze the chemical composition, energy-dispersive X-ray (EDX) spectroscopy using the XFlash detector 6|30 was done with an acceleration voltage of 20.0 kV and a probe current of 4.0 nA. Quantification was done using the Cliff-Lorimer equation. The intensities of the element-specific X-ray lines were determined by using Gaussian functions. The k-factors were calculated using the Bruker software. The results were normalized to Cu. In the case of thin films on substrates, the spectrum can also contain signals from the substrate as a result of the large interaction volume when using high acceleration voltages in SEM. For example, the In L line from CuInS_2_ and the Sn L line from the substrate FTO (SnO_2_:F) overlap and complicate the quantification of In (compare also our recent publication) [38]. Nevertheless, EDX measurements have been performed in the SEM at 20 kV acceleration voltage but In is not considered for the analysis and only the ratio between Cu and S is given (stoichiometric ratio Cu:S for CuInS_2_ should be 1:2).

The film thicknesses were measured by focused ion beam (FIB) sectioning on a FEI Helios Nanolab 600. Cuts were performed at sample areas coated with conductive silver paint to avoid destruction of the film surface.

To conduct UV-vis measurements of the CuInS_2_ films a Perkin Elmer Lambda 800 in transmission mode has been used. Spectral range was from 260 nm to 900 nm with a step size of 1 nm. From the UV-vis data band gaps were calculated using the Tauc method for direct band gap semiconductors [39]. The energy was plotted vs. (energy∙absorption)^2^ and the first linear slope was fitted and the intersection with the *x*-axis calculated.

For in-depth characterization of the films (scanning), transmission electron microscopy ((S)TEM) was used. Measurements were performed on a FEI Titan Themis 300 (S)TEM at 300 kV acceleration voltage. The (S)TEM is equipped with a C_S_ probe corrector, a Gatan Quantum ERS energy filter, and a Super X-EDX detector from Bruker. Electron diffraction data, calibrated with the help of a Si standard, were evaluated by comparing the results to literature data. (S)TEM scratch samples have been prepared to avoid an influence of the sample preparation on the crystallinity and composition of the investigated films (compare a recent publication of our group) [38]. As mentioned for EDX in SEM, quantification was done using the Cliff-Lorimer equation with the help of the Bruker software and normalizing the results relative to Cu. In STEM mode, several EDX maps have been recorded (≈6 maps per sample) and quantification of the Cu:In:S ratio was done on ≈10 areas of each map with each area ≈ 100 nm^2^ and calculating the average value.

## 3. Results

### 3.1. Reaction Time

**Figure 1:**
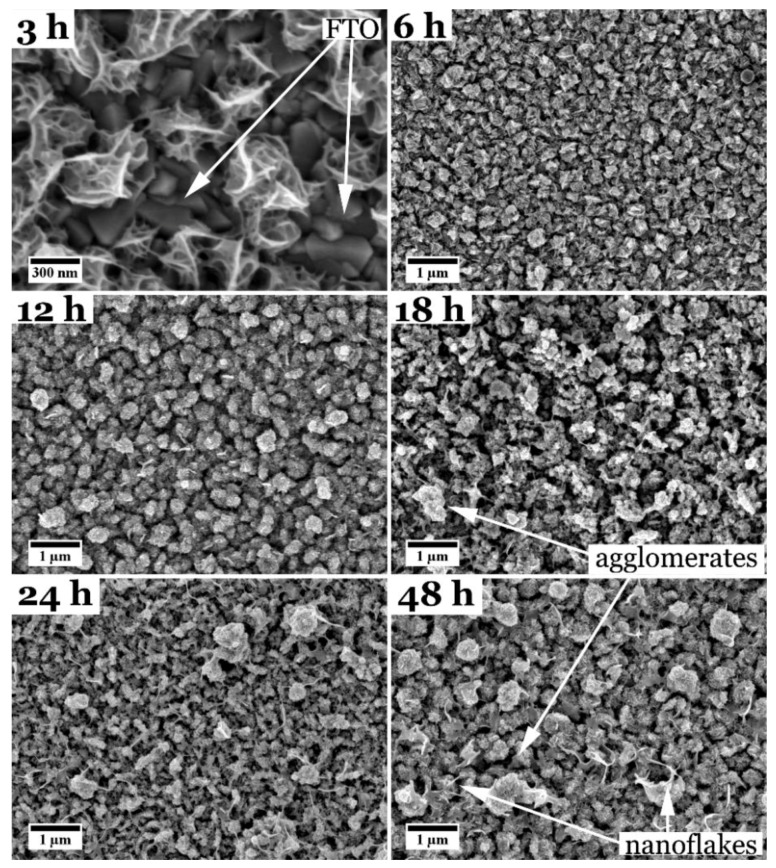
Top-view SEM images of CuInS_2_ films synthesized with l-cysteine for different reaction times at 150 °C. The time varied between 3 h and 48 h. Please note a different scale bar for a reaction time of 3 h (film_3 h).

Top-view secondary electron SEM images of CuInS_2_ films, solvothermally synthesized for different reaction times at 150 °C, are shown in Figure 1 and in the Supporting Information (Figure S1). At first sight, all the films show a very similar surface topology. Only film_3 h, synthesized with the shortest reaction time of only 3 h at 150 °C, seems to be composed of individual agglomerates that grow on the FTO surface. Between these small agglomerates, the substrate is still visible, indicating an incomplete coverage after 3 h of solvothermal reaction (see Figure 1). With increasing reaction time, the agglomerates seem to grow laterally until they cover the underlying FTO substrate completely and form a compact CuInS_2_ layer (for cross-sectional views see later images and the supporting information, Figure S2). After the compact layer has formed, more CuInS_2_ agglomerates deposit on top of it. For some of the films, small nanoflakes growing out of the agglomerates can be observed (exemplarily marked in Figure 1). Nevertheless, large changes in the surface morphology of the films cannot be seen. From 6 h reaction time, the FTO substrate is not visible anymore in top-view and no cracks or delamination of the film is observed in cross-sectional SEM micrographs (see Figure S2 in the supporting information and also Figure 4a,b), implying a good homogeneity and adhesion of the films to the substrate.

Measurement of the film thicknesses of the films via FIB is difficult, as the films consist of a compact CuInS_2_ layer and outgrowing agglomerates. This problem is demonstrated exemplarily with the help of a FIB cross sectional cut, shown in Figure S2 (can also be seen in Figure 4a,b for a (S)TEM cross sectional sample). However, for all films, the thickness of the compact layer was determined to be in a relatively small range around 400 nm and is therefore in the same size regime.

**Figure 2:**
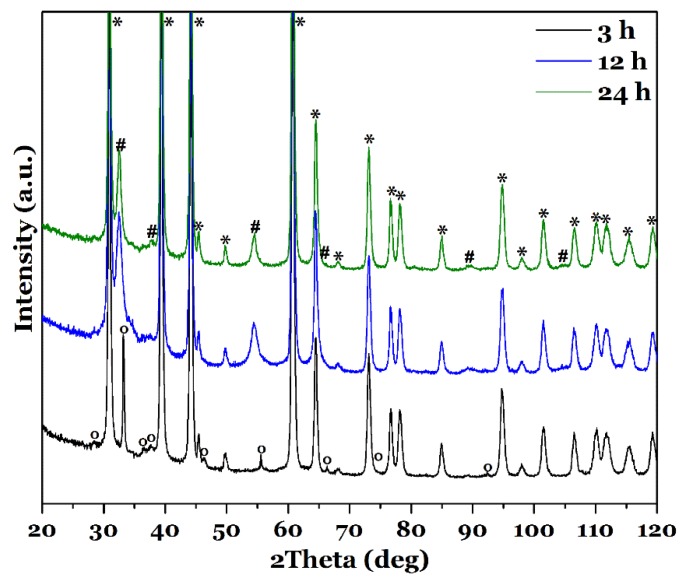
XRD pattern of CuInS_2_ films synthesized with l-cysteine for different reaction times, 3, 12 and 24 h, at 150 °C. Signals stemming from FTO are marked with *, the ones originating from CuInS_2_ with #, reflections from CuCl with °. The ° reflections could also stem from InS.

Figure 2 shows exemplary XRD pattern of the films grown for 3, 12 and 24 h at 150 °C. The XRD pattern of the films synthesized for the other reaction times and of the pure FTO substrate are shown in the supporting information (Figures S3 and S6). Besides the strong reflections of the substrate FTO, (marked with * in Figure 2) all films show some more distinct signals. These reflections can be indexed according to tetragonal CuInS_2_ in its Chalcopyrite modification (Figure 2, marked with # and compared to literature data [40]) for the films synthesized with a reaction time of at least 6 h. However, Cu^I^ and Cu^II^ sulfides possess very similar *d-*values as CuInS_2_ and can therefore not be excluded by XRD data alone [41,42,43]. Additionally, amorphous phases could be present. Only film_3 h displays different reflections compared to the other films, which possess pure CuInS_2_. These reflections (marked with °) can be assigned to cubic CuCl [44] and orthorhombic InS [45]. Due to the EDX measurements, described below, the presence of InS can be excluded as there is nearly no In detectable in the film as described later.

When comparing the sharp FTO reflections with the ones from tetragonal CuInS_2_ it becomes clear that the latter is rather broad, indicating a small crystal and/or domain size in the films. Applying the Scherrer equation [37] to the most intense reflections (112) and (204) of CuInS_2_ allows to estimate crystal sizes that are on average 9.0 ± 1.0 nm (Table 1). For film_3 h, in comparison, the distinct signals are relatively sharp, and using the (111) and (220) reflections of CuCl gives a crystal size of ≈39 nm for this phase. This value is three times higher than the largest calculated crystallite size of the CuInS_2_ films.

EDX measurements in SEM, as mentioned before, show not only signals from Cu, In and S, but also from the substrate (Sn, Si, O from FTO and glass) due to the large interaction volume. This is discussed in more detail in our previous publication [38]. The overlap between the In L and Sn L line make the quantification of In difficult. This is the reason why for SEM EDX measurements only the Cu:S ratio is given and In is not included. Furthermore, all the SEM EDX spectra show also signals from the conductive coating (Au, Pd) and Cl from the InCl_3_ precursor.

For film_3 h, a very high amount of Cu and Cl is measured with a lower amount of S and nearly no signal from In. This proves the existence of CuCl as observed from the XRD pattern (see Figure 2). However, an InS phase, which could also explain the reflections in the XRD pattern, is not present due to the very low In amount. Possible other phases might be, e.g., a strongly distorted CuS [41]. This will be described in more detail later. As can be seen (Table 1), the sulfur ratio detected for the films is varying, but always close to the stoichiometric Cu:S ratio of CuInS_2_ of 1:2, except for reaction times of 3 h and 6 h. Higher amounts of S can be attributed to e.g., incomplete cleaning of the synthesized films with water and therefore remaining precursors/amorphous side products on the film surface. To quantify also the In amount via EDX (S)TEM measurements were performed, which are described later.

**Figure 3:**
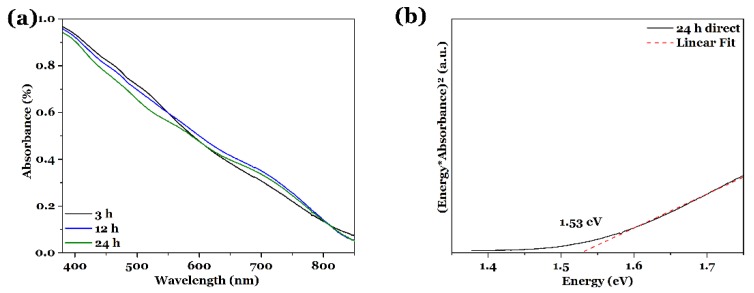
(**a**) UV-vis spectra of CuInS_2_ thin films on FTO substrate, synthesized solvothermally with l-cysteine for 3, 12 and 24 h at 150 °C. (**b**) Exemplary Tauc plot for direct semiconductors for film_24 h, indicating a band gap of 1.53 eV.

All films, synthesized with reaction times from 3 to 48 h, show a similar, strong absorption behavior over the whole visible spectrum, as exemplarily shown for film_3 h, film_12 h and film_24 h in Figure 3 (the UV-vis spectra from the other samples can be found in the supporting information, Figure S4). The absorption is influenced by the film thickness and also by light scattering on film structures or on the interface to the substrate. Since the film thicknesses of our films varies slightly but stays in the same size regime (≈400 nm), this should not influence the absorption drastically. However, our films possess a rough surface structure (compare Figure 1) with larger agglomerates on top, which can scatter the light and ‘increase’ the absorption. Additionally, in areas with less agglomerates, the absorption is lower due to a smaller effective film thickness. Furthermore, crystal structure and composition can influence the total absorption of the measured films.

The optical band gaps were calculated from the UV-vis data and the values are summarized in Table 1. In Figure 3b, an exemplary Tauc plot for film_24 h is shown. The smallest band gap of 1.44 eV is observed for film_48 h and the largest one with a value of 1.55 eV for film_3 h. All these values are close to the reported band gaps for CuInS_2_ bulk material and nanostructures [21,46]. Variations in the band gap values can also be affected by the chemical composition, the film thickness and structure, as well as defects in the crystal structure, as mentioned above for the absorption behavior. Due to the solvothermal synthesis of our films and the small crystal size, a large number of defects, i.e., grain boundaries, are present, which could also lead to sub-band gap excitations [47,48,49]. Although the band gaps seem to vary (compare Table 1) no correlation can be drawn between the band gap value and, e.g., the crystallite size or chemical composition. The average band gap for the films grown for 6 to 48 h is 1.50 ± 0.04 eV. The standard deviation is relatively small, so that the band gaps can be considered as similar for all reaction times. This will be discussed in more detail later.

**Figure 4:**
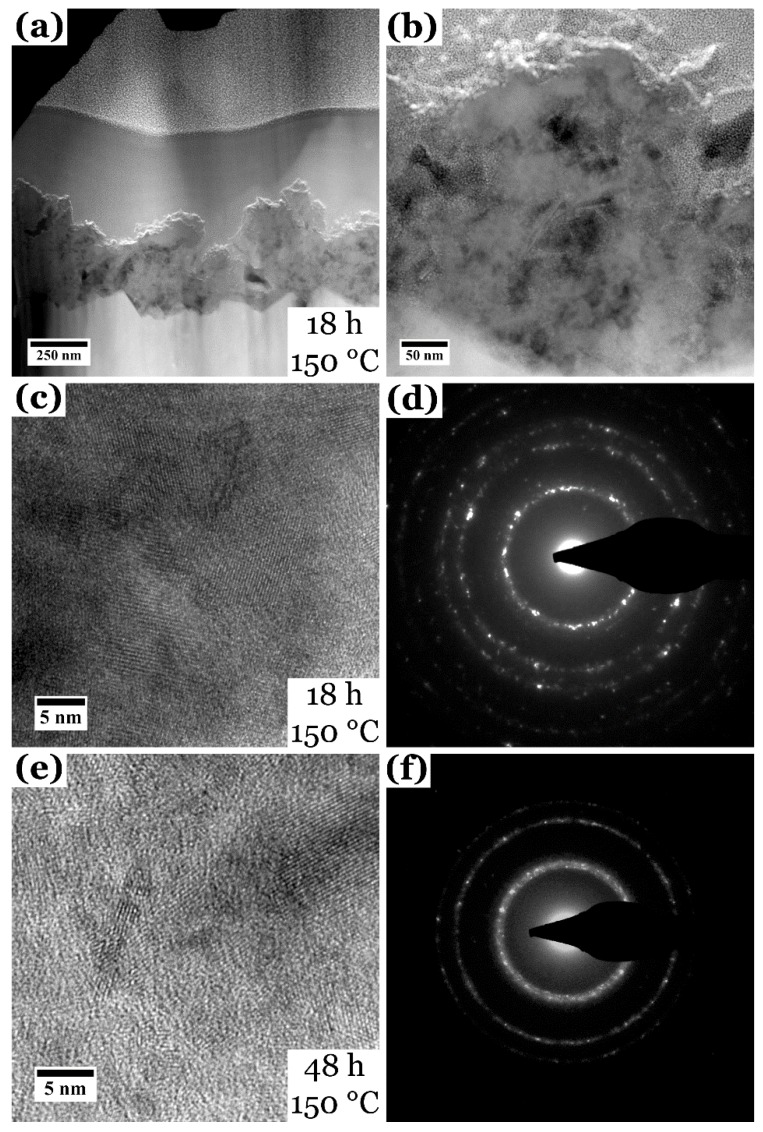
(**a**,**b**) cross sectional HAADF STEM images of a lamella prepared from film_S [32,38], displaying the vertical structure of the film. (**c**,**d**) HR TEM image and according electron diffraction pattern of film_S, and (**e**,**f**) from film_48 h.

To investigate and compare the films grown at different reaction times in more detail, (S)TEM investigations have been performed. For the STEM EDX data, the In quantification is not problematic because the influence of the substrate FTO (and therefore the signal from Sn) can be neglected. Out of high-resolution HR TEM images, the crystal size and structure was extracted and compared to the one obtained by XRD. The results of the (S)TEM measurements on film_S [32,38] and film_48 h are shown in Figure 4, a TEM image and according diffraction pattern taken from film_3 h is shown in the supporting information, Figure S5, but also discussed in the following.

Figure 4a,b show the cross-sectional view of a focused-ion beam prepared lamella in STEM, demonstrating the vertical structure of the CuInS_2_ film grown for 18 h at 150 °C. The film consists of a compact layer close to the FTO substrate with larger agglomerates growing on top of the film [32,38]. As all the other films, grown for different reaction times, display very similar topologies in SEM, it can be concluded that they also display very similar vertical structures. The HR TEM image and electron diffraction pattern in Figure 4c,d, also taken from film_S, prove the good crystallinity of the nanoparticle film and the tetragonal CuInS_2_ modification [32,38]. The crystal size of the nanoparticles determined from the HR TEM images is 5.3 ± 2.2 nm and therefore smaller than the one determined with XRD (9.4 nm, compare Table 1). EDX quantification for film_S resulted in Cu 24 ± 2 at %, In 25 ± 2 at % and S 51 ± 3 at %, giving a Cu:In:S ratio of 1.0:1.0:2.1 [32]. This ratio is close to the stoichiometric value.

As for film_S, the HR TEM image and electron diffraction pattern for film_48 h reveal a good crystallinity of the solvothermally synthesized CuInS_2_ film in the tetragonal Chalcopyrite modification (Figure 4e,f). Again, the film is composed of many agglomerated nanoparticles with grain sizes of 5.4 ± 2.3 nm, smaller than the value calculated from the XRD data (Table 1). The reason why the determination of the crystallite size leads to different values in XRD and TEM will be discussed later. EDX measurements on various areas gave Cu 24 ± 2 at %, In 25 ± 1 at % and S 52 ± 1 at %, resulting in a Cu:In:S ratio of 1.0:1.0:2.2. This is also very close to a stoichiometric composition of CuInS_2_.

Only the film grown for 3 h showed single agglomerates of nanoparticles on the FTO substrate (see Figure 1) and strong reflexes of CuCl in the XRD data. HR TEM and electron diffraction pattern (Figure S5a,b) confirm that these nanoparticles are crystalline. The crystallite sizes determined from HR TEM images resulted in a minimum value of ≈4 nm and a maximum crystal size of 36 nm; the latter was also obtained out of the XRD spectrum. The electron diffraction pattern can be indexed according to cubic CuCl [44] and orthorhombic InS [45]. However, due to very similar *d-*values of other possible products, e.g., In_2_S_3_ or CuS, and the possibility of lattice distortion of these phases caused by intercalation of impurity atoms, it is very difficult to determine the unambiguous phases. EDX measurements and quantification have been performed for Cu, In, S and also Cl. The quantification led to 48 ± 5 at % Cu, 2 ± 2 at % In, 10 ± 8 at % S and 39 ± 6 at % Cl, which gives a ratio of Cu:Cl of 1:0.8. This is close to the stoichiometric Cu:Cl ratio of 1:1 for CuCl. The existence of an InS phase in film_3 h can be excluded because of the very low Indium amount.

### 3.2. Reaction Temperature

**Figure 5:**
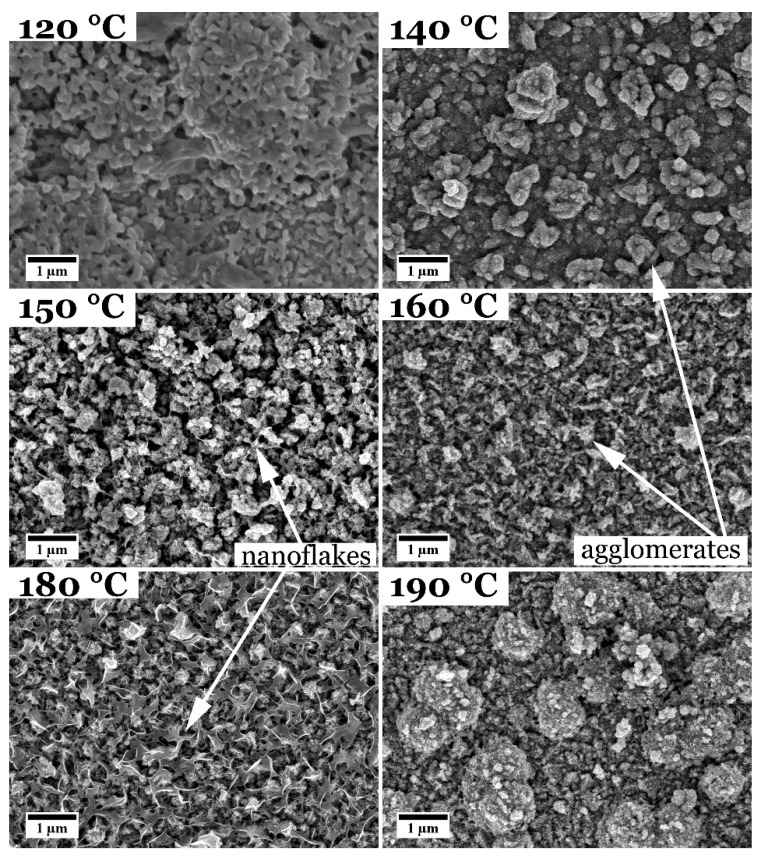
Top-view SEM images of CuInS_2_ films synthesized with l-cysteine at different reaction temperatures for 18 h. The temperature for the films shown was varied between 120 and 190 °C.

Figure 5 shows an overview over the CuInS_2_ samples solvothermally grown with l-cysteine at different reaction temperatures for 18 h. Film_100 °C is shown in the supporting information (Figure S6). A reaction temperature of 100 °C seems to be not sufficient to grow a film. Only the pure FTO substrate is found in SEM and XRD (Figure S6). Except for film_100 °C and film_120 °C, all the films have the same appearance when studied in top-view as the films synthesized at 150 °C with different reaction times (see Figure 1). The films consist of a compact nanograined film with agglomerations of nanoparticles on top, which vary in size and density. Film_120 °C, on the other hand, looks different. The film seems to consist of nanoparticles, too, but with a smoother shape and has a white appearance on the FTO substrate when inspected by eye, while all other films are brownish. For film_180 °C, although similar to the other CuInS_2_ films, a higher number of nanoflakes, which grow out of the film, and agglomerates can be observed. This can also be seen in a more distinct manner for other films (compare Figure 1). The film with the highest reaction temperature (190 °C) displays very large, round-shaped agglomerates. All films again show no cracks or delamination from the substrate.

The film thickness of these films has also been investigated by FIB cross sectional cuts. Again, the film thickness of all films is around 400 nm. As mentioned before, the measurement of the film thickness is difficult because of the structure of the film (Figure S2 and HAADF STEM image in Figure 4a).

**Figure 6:**
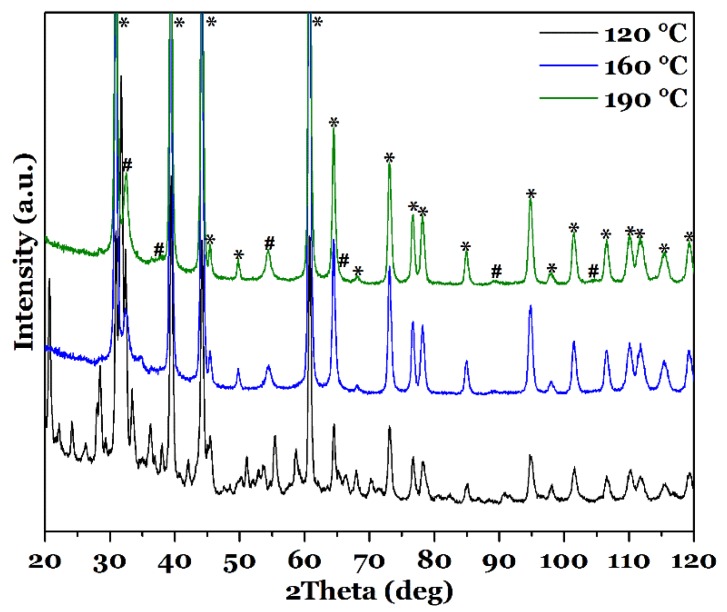
XRD pattern of CuInS_2_ films synthesized with l-cysteine at different reaction temperatures, 120, 160 and 190 °C, for 18 h. Signals stemming from FTO are marked with *, the ones originating from CuInS_2_ with #.

XRD patterns obtained from the films synthesized at different reaction temperatures for 18 h are shown in Figure 6, exemplarily for 120, 160 and 190 °C, and in the supporting information, Figures S6 and S7, for the other temperatures. For film_100 °C, as already observed in the SEM, only the pure FTO substrate leads to signals in the XRD pattern. All peaks are in accordance to literature data [50]. This implies that a reaction temperature of 100 °C is not sufficient for the growth of CuInS_2_. In addition, the XRD pattern of the sample grown at 120 °C (Figure 6, black) does not correspond to CuInS_2_. The pattern shows a lot of reflections, which cannot be indexed unambiguously by one crystalline phase. This means that several different crystalline species are formed for this reaction conditions. It might also be that the film consists of not-reacted precursor salts or preliminary formed complexes. However, with further increase of the reaction temperature, CuInS_2_ in tetragonal Chalcopyrite modification is formed. As mentioned before, the presence of side products cannot be excluded fully because of very similar *d-*values of possible compounds, e.g., copper sulfides. Amorphous phases cannot be excluded as well. However, within the detection limit, a pure CuInS_2_ phase is formed for all films.

The CuInS_2_ peaks are again rather broad and crystal sizes between 7.9 nm for film_140 °C and 11.4 nm for film_180 °C were estimated from the XRD data. On average, the crystal/domain sizes lay also in the range of 10 nm as observed for the CuInS_2_ films grown for different reaction times at 150 °C (compare Table 2).

Analogous to the study of different reaction times, also for the films grown at different reaction temperature, EDX measurements have been performed in the SEM but only the Cu:S ratio is given.

As expected, film_100 °C does not give any signal of Cu, In or S in the EDX spectrum, as it is only the plain FTO substrate and no film has grown on top. Film_120 °C shows only a very little amount of copper (Cu:S 1.0:3.9) but large amounts of from Sn and O (FTO substrate). From film_140 °C on the films show a nearly stoichiometric ratio between Cu and S. An increase of the reaction temperature to 180 °C gives a ratio of Cu to S of 1.0:1.6 (Table 2). The decreased amount of sulfur is even more pronounced for a reaction temperature of 190 °C (Cu:S of 1.0:1.4). However, as the XRD pattern in Figure 6 only shows signals from CuInS_2_ in the tetragonal Chalcopyrite modification, the reduced S amount might be caused by e.g., amorphous side products, which have not been rinsed away.

**Figure 7:**
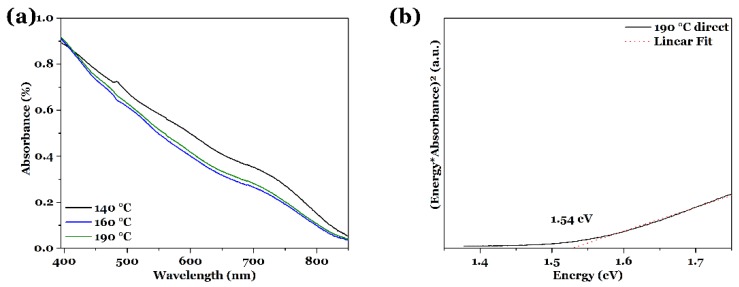
(**a**) UV-vis spectra of CuInS_2_ thin films on FTO substrate, synthesized solvothermally with l-cysteine at different reaction temperatures for 18 h. (**b**) Exemplary Tauc plot for direct semiconductors for film_190 °C, indicating a band gap of 1.54 eV.

All the films show a good absorption behavior over the whole visible spectrum (see Figure 7a for film_140 °C, film_160 °C and film_190 °C, the other UV-vis spectra are shown in the supporting information, Figure S8). As described before, fluctuations in the total absorption can be caused by variations of the sample surface, relative thickness, and scattering of light. Because all the films show a similar crystal size (determined with XRD) and film thickness, the absorption is in the same order of magnitude.

Figure 7b shows one exemplary Tauc plot to determine the band gap for film_190 °C; the other calculated band gaps are summarized in Table 2. The smallest band gap is found for film_S (1.47 eV) [32], while the largest one is given for a reaction temperature of 180 °C, with a value of 1.55 eV. All the other band gap values lay in between these values and are very close to band gaps reported in literature for CuInS_2_ [21,46]. Again, variations in the band gap can be induced by the chemical composition, the film thickness, and structure, as mentioned already above. The average band gap gives 1.52 ± 0.03 eV, which also displays a small standard deviation and therefore the band gaps are in the same size regime. The impact of these fluctuations in the band gap values will be discussed later.

**Figure 8:**
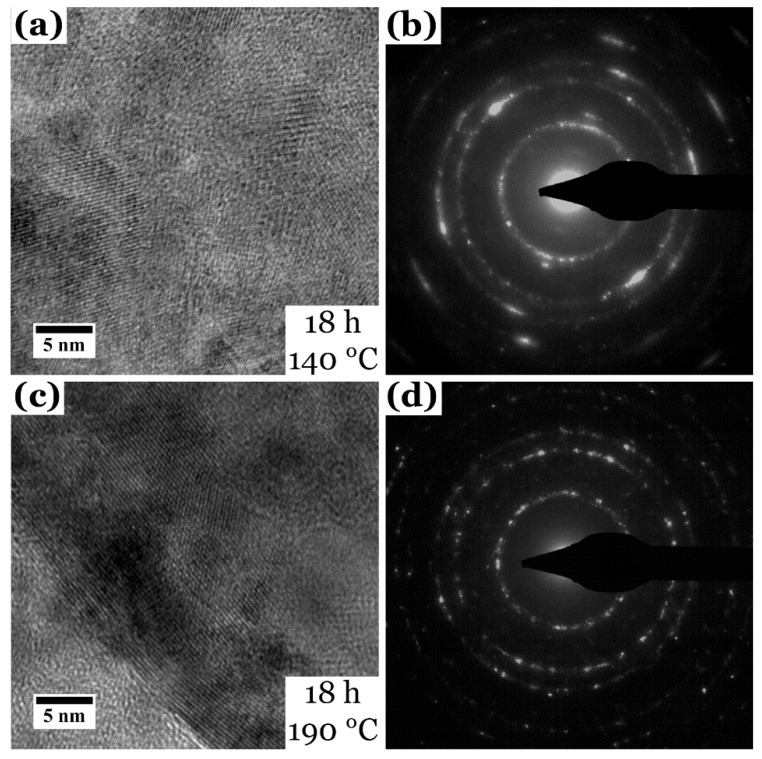
(**a**) HR TEM image and (**b**) corresponding electron diffraction pattern of film_140 °C. (**c**) HR TEM image and (**d**) corresponding electron diffraction pattern of film_190 °C.

TEM measurements on film_140 °C show a good crystallinity of the film (Figure 8a,b) and small crystallites with an average size of 5.7 ± 2.0 nm (XRD 7.9 nm, compare Table 2). The size of the crystals is in the same magnitude as for film_S [32] and film_48 h (compare also Figure 4 and Table 1); the deviation between TEM and XRD grain size determination will be discussed later. The electron diffraction pattern in (b) also proves the tetragonal Chalcopyrite modification of CuInS_2_. STEM EDX quantification gives Cu 23 ± 1 at %, In 25 ± 1 at % and S 52 ± 1 at %, resulting in a Cu:In:S ratio of 1.0:1.1:2.2, which is very close to a stoichiometric CuInS_2_ ratio.

The (S)TEM investigations on film_190 °C (Figure 8c,d) give similar results. The film is also well crystallized and displays the tetragonal CuInS_2_ modification. Crystallite size was determined to be 10 ± 5.0 nm, which is larger than for the lower temperatures but close to the crystal size determined with XRD (≈9.8 nm). Additionally, the reflections in the diffraction pattern of film_190 °C appear much sharper than for film_140 °C (Figure 8b vs. Figure 8d), hinting also at a larger crystallite size. The quantitative EDX measurements in STEM mode show Cu 26 ± 1 at %, In 25 ± 1 at % and S 49 ± 1 at %, resulting in a Cu:In:S ratio of 1.0:1.0:1.9. This is also very close to stoichiometry of CuInS_2_.

## 4. Discussion

When comparing the top-view SEM images of the samples synthesized with strongly varying reaction time and temperature, they all appear very similar. Most films are composed of a dense film with outgrowing agglomerates, also built up of nanoparticles, on top. This can also be seen in cross-sectional SEM and STEM images. The film thickness of the different films is roughly in the same size regime of ≈400 nm. This can be explained by the use of the same precursor concentrations for all the films, which was found to be the dominating parameter in controlling the film thickness [32]. When all available precursors are consumed, neither time nor temperature have an impact on the thickness of the grown CuInS_2_ film. Only changing the number of precursor molecules would change the film thickness. However, another possible explanation for this fact is the development of a thermodynamic equilibrium between grown film and free CuInS_2_ nuclei. Only adding more precursor would shift the equilibrium to an increase in film thickness.

For all synthesis conditions, a pure tetragonal Chalcopyrite phase of CuInS_2_ can be observed in the XRD data, except for film_3 h, film_100 °C and film_120 °C. Applying the Scherrer equation [37] to the most intense peaks of the pattern gave an average crystal/domain size of ≈10 nm. Crystal sizes determined with TEM for film_S [32], film_48 h and film_140 °C give, by contrast, smaller crystal sizes of ≈5 nm with the exception of film_190 °C, where both methods led to similar crystal sizes (≈10 nm). This might be related to the fact that the films are buildup of two areas—a dense, nanograined film and overlying agglomerates of nanoparticles. Due to the global nature of XRD, the calculation of the crystallite size takes into account both parts of the film structure, resulting in an overall higher crystal size. In contrast, TEM allows the determination of the crystal size at a very local scale and the here used scratch TEM samples are most likely representing mainly the agglomerates, as they can be removed from the substrate easier. As a consequence, we conclude that our CuInS_2_ thin films display larger crystal sizes in the more-dense film close to the substrate (measured with XRD), while the agglomerates on top of this film are formed of smaller crystallites (measured with TEM). Only for film_190 °C, the crystal size determined with TEM is close to the XRD measurements, which can mean that higher temperatures lead to larger crystal sizes. Nevertheless, all CuInS_2_ thin film samples display a good crystallinity and the tetragonal Chalcopyrite modification of CuInS_2_. Along with EDX measurements, it can be concluded that starting from a reaction time of 6 h and a reaction temperature of 140 °C, pure CuInS_2_ thin films without any impurities or side products can be grown solvothermally with the help of l-cysteine. The reason for the indeterminable amount of side products like CuS, Cu_2_S or In_2_S_3_ is the use of a high enough l-cysteine to Cu precursor ratio of 2.5:1 [32,51] as well as the complex formation between Cu, In and l-cysteine (and cystine) [35,36,52,53] as will be described below.

All the films show a very good absorption behavior in the visible regime and their band gaps, calculated with the help of the Tauc method, lie in the range between 1.46 and 1.55 eV, which is very close to the band gap for the bulk CuInS_2_ [21] and has also been reported for CuInS_2_ nanostructures [46]. As obvious from Table 1 and Table 2 and mentioned before, the band gap values of the different films are varying and show no direct relation to the crystal size and/or chemical composition. An average value calculated from the band gaps of film_6 h to film_48 h as well as film_140 °C to film_190 °C results in 1.52 ± 0.04 eV. The small standard deviations show that the band gap variations are small and can be caused by e.g., slight variations in the chemical composition or the local crystallinity, which can be caused e.g., by amorphous side products, etc. Summarized, the absorption properties and band gap values, as well as the good crystallinity, make the CuInS_2_ thin films a very interesting material for solar driven applications. Changes of the band gaps in the here observed regime should not have any influence on use in applications. A calculation of the theoretical efficiencies based on the band gap values show that, considering single-junction solar cells, a high efficiency of ≈30% can be reached in a band gap range from ≈1.0–1.6 eV [54,55]. This means that for a solar-driven application, the observed band gap values are excellent. Equally important is the ability to absorb most of the sun light, which is true for CuInS_2_.

Solvothermal reaction—and chemical reactions in general—are governed by many factors, but most important are the thermodynamic ones, pressure, and temperature, followed by reaction time. In this work, we varied the reaction time at constant temperature, and the reaction temperature for constant time intervals. In the case of a reaction time of 18 h but increasing reaction temperature, the pressure inside the reaction vessel should increase accordingly. However, the consideration in the case of a fixed reaction temperature but changing time is more complex. In general, for an increase of reaction time at fixed temperature, no significant increase in pressure is to be expected—except for the evolution of gaseous products during the reaction, which is not assumed for the CuInS_2_ reaction as presented here. Nevertheless, for very short reaction times, the temperature inside the autoclave is lower than targeted and a therefore lower pressure is expected.

For film_3 h, mainly a cubic CuCl phase could be observed in the XRD and electron diffraction pattern. The film itself is very thin and the FTO substrate is visible between the agglomerates. EDX measurements in STEM mode reveal mainly Cu and Cl with only little indium and sulfur contents in the film. This shows that a reaction time of 3 h at 150 °C is not sufficient to form CuInS_2_. The formation of CuCl instead can be explained as follows: Although a reaction temperature of 150 °C is chosen, the autoclave might not reach this temperature during 3 h of reaction time. For a short reaction time (i.e., lower temperature, lower pressure inside the autoclave) the decomposition of l-cysteine and cystine proceeds with a kinetically low rate, leading to (1) the metal ions In^3+^ and Cu^+^ still stabilized in complexes and (2) no S^2−^ ions are available. Instead, a lot of free Cl^−^ ions are accessible due to the solvation of the InCl_3_ precursor salt. A recrystallization of InCl_3_ is very unlikely because of an enthalpy of formation of 537 kJ/mol [56], which exceeds the one of In_2_S_3_ of −346 kJ/mol [56]. However, as mentioned before, due to the low temperature/short reaction time, no sulfur ions are available. Therefore, only CuCl with an enthalpy of formation of −137 kJ/mol [56] is thermodynamically likely to grow.

For film_100 °C, only the pure FTO substrate was obtained. It is obvious that a reaction temperature of 100 °C, although kept for 18 h, and an according low pressure inside the autoclave, is not sufficient to grow CuInS_2_ or any other crystalline compound on the substrate. The formed Cu^+^ and In^3+^ complexes with l-cysteine and cystine are still stable at this temperature and a thermal decomposition is not taking place. Therefore, no S^2−^ and metal ions are released and ready to react. Additionally, the overall solubility of the precursor salts is not promoted at low temperature and pressure, and only a little amount is available in the solution.

When increasing the reaction temperature to 120 °C, a film forms, consisting of nanoparticles, probably embedded in an amorphous matrix. The XRD data show a lot of signals, arising from crystalline compounds, which means that the reaction conditions are sufficient to form crystalline compounds. However, an unambiguously assignment of all emerging signals is not achievable. Possible compounds are l-cystine, l-cysteine, their Cu and In complexes, Cu sulfides like Cu_2_S, Cu_1.8_S or CuS, indium sulfides, e.g., In_2_S_3_ or modifications of these phases. It is also possible that unchanged precursor materials like InCl_3_ or CuSO_4_∙5H_2_O are still present for this reaction conditions. As observed for film_3 h, the formation of CuCl is also possible.

The reaction mechanism of CuInS_2_ out of CuSO_4_∙5H_2_O, InCl_3_ and l-cysteine can be described as follows: as known, l-cysteine is oxidized to cystine while Cu^2+^ is reduced to Cu^+^ [32,51,53]. This Cu^+^ and In^3+^ can be coordinated and stabilized by the chelating agents l-cysteine and cysteine [57]. The release of the metal ions is therefore very slow. As can be learned from a reaction at 100 °C for 18 h, at the chosen precursor concentrations (Cu 0.2 mol, In 0.2 mol, l-cysteine 0.5 mol, precursor ratio Cu:In:S 1:1:2.5) no free metal or sulfur ions exist in the solution. However, reaching a certain temperature/pressure (>120 °C), the organic molecules l-cysteine and l-cystine begin to decompose and release S^2−^. When the decomposition of the sulfur source starts and S^2−^ is released, (InS_2_)^−^ can be formed [35,36,52], but also copper sulfides are possible side products. From previous investigations [32] with higher l-cysteine contents (Cu:In:S precursor ratio 1:1:4), it is known that during a reaction at 150 °C for 18 h, a compact CuInS_2_ bottom layer with an outgrowing In_2_S_3_ top layer is formed. Accordingly, for a short reaction time (3 h) at 150 °C, In_2_S_3_ should be formed, which is not the case as described above. Here, the reason is a thermodynamically favored formation and stabilization of Cu^+^ in a chloride as no sulfur ions are present. Additionally, this gives rise to the conclusion that the combination between (InS_2_)^−^ and Cu^+^ to form CuInS_2_ is the main bottleneck in the reaction and rather slow, which hints at a higher importance of the reaction time compared to the reaction temperature. However, as obvious from film_100 °C and film_120 °C, the reaction temperature is important in the formation of CuInS_2_. If the temperature of the reaction is not high enough, no reaction will occur (film_100 °C) or a mixture of compounds will grow (film_120 °C). However, temperatures above 120 °C allow the formation of pure CuInS_2_ without obvious side products, as described above.

Our results are different from Kharkwal et al. [52] They synthesized CuInS_2_ nanoparticles solvothermally using CuCl, InCl_3_ and thiourea at 150 °C for different reaction times (2 to 48 h). They observed small nanoparticles of ≈5 nm for 2 h reaction time and larger nanoparticles (up to ≈27 nm) for 48 h. All nanoparticles showed a pure tetragonal CuInS_2_ and with an increase in the particle size, the band gap decreased. Furthermore, with increasing reaction time, their nanoparticles evolved from agglomerates to flower-shaped structures. Kharkwal et al. ascribed the phase purity of their nanoparticles to the formation of Cu and In complexes with thiourea as we ascribe them to the formation of l-cysteine complexes. However, in our case, the crystallite size does not change with reaction time and a reaction time of 3 h is not sufficient to produce CuInS_2_. This can be related to the different sulfur and copper precursors. In addition, in our case, the Cu^2+^ of the precursor has to be reduced to Cu^+^ before it is usable in the synthesis of CuInS_2,_ while Kharkwal et al. [52] directly used a Cu^+^ salt.

Zhuang et al. [58] prepared CuInS_2_ thin films on FTO substrates as well. They used CuCl_2_, In(NO_3_)_2_, thiourea, oxalic acid and hexadecyl trimethyl ammonium bromide (CTAB) in ethanol at 200 °C and reaction time was varied (1, 4, 8 and 20 h). For a low thiourea concentration, they observed an increase in film thickness with increasing reaction time (saturation after 8 h of reaction) and an evolution in the surface topology. For a higher concentration, the films are always composed of a close packed microsphere layer, while only the diameter and packing density changed with time. All the samples displayed the tetragonal Chalcopyrite modification of CuInS_2_. In our synthesis, the influence of the reaction time on film thickness or morphology is neglectable.

To summarize the comparison with literature, it seems that our synthesis procedure using l-cysteine as sulfur source is a very robust route, as it yields CuInS_2_ films on FTO substrates with excellent properties over a wide reaction temperature and time range.

## 5. Conclusions

To conclude, we were able to demonstrate that the green synthesis of pure, nanocrystalline CuInS_2_ thin films on FTO substrates via a solvothermal, non-toxic l-cysteine assisted synthesis approach is possible and stable over a wide reaction time and temperature range. Our systematic study showed that the synthesis of CuInS_2_ films with a thickness of ≈400 nm, a good crystallinity and band gaps in the range of 1.46 to 1.55 eV is independent of the exact reaction temperature and time, as long as the reaction temperature is above 140 °C and the reaction time longer than 6 h. All the results can be explained by the presented, refined reaction mechanism and the stability of initially formed precursor complexes of Cu and In complexes with l-cysteine and cystine, which provides the basis for the advantage of using l-cysteine compared to other sulfur sources. The facile and robust solvothermal synthesis of CuInS_2_ using l-cysteine is therefore suggested as a possible route for up-scaling. Due to their excellent properties, the films are also viewed as possible candidates for solar driven applications like solar cells or water splitting.

## Figures and Tables

**Table 1:** Summary of crystal size, determined with XRD, normalized elemental composition (Cu:S for SEM, Cu:In:S for STEM measurements) and band gap of the CuInS_2_ films synthesized with l-cysteine for different reaction times at 150 °C. Values of film_3 h are not included in the average value in the last line.

	Crystal Size XRD (nm)	EDX SEM Cu:S (Normalized)	EDX TEM Cu:In:S (Normalized)	Band Gap UV-vis (eV)
film_3 h	39 ± 4	---	Cu:Cl 1.0:0.8 Cu:In:S 1.0:0.1:0.2	1.55
film_6 h	6.6 ± 1.4	1.0:1.3	---	1.54
film_9 h	11.0 ± 0.6	1.0:1.6	---	1.46
film_12 h	7.9 ± 1.0	1.0:2.1	---	1.54
film_15 h	8.5 ± 0.6	1.0:2.4	---	1.50
film_S [32]	9.4 ± 1.0	1.0:2.5	1.0:1.0:2.1	1.47
film_21 h	8.9 ± 1.0	1.0:2.3	---	1.51
film_24 h	11.0 ± 0.5	1.0:1.7	---	1.53
film_48 h	8.7 ± 1.0	1.0:1.9	1.0:1.0:2.2	1.44
Ø	9.0 ± 1.0	1.0:2.0 ± 0.4	---	1.50 ± 0.04

**Table 2:** Summary of crystal size, determined with XRD, normalized elemental composition (Cu:S for SEM, Cu:In:S for STEM measurements) and band gap of the CuInS_2_ films synthesized with l-cysteine at different reaction temperatures for 18 h. Values of film_120 °C are not included in the average value in the last row.

	Crystal Size XRD (nm)	EDX SEM Cu:S (Normalized)	EDX TEM Cu:In:S (Normalized)	Band Gap UV-Vis (eV)
film_120 °C	---	1.0:3.9	---	1.51
film_140 °C	7.9 ± 0.5	1.0:1.9	1.0:1.1:2.2	1.52
film_S [32]	9.4 ± 0.6	1.0:2.5	1.0:1.0:2.1	1.47
film_160 °C	9.2 ± 1.0	1.0:1.7	---	1.54
film_180 °C	11.4 ± 1.0	1.0:1.6	---	1.55
film_190 °C	9.8 ± 0.6	1.0:1.4	1.0:1.0:1.9	1.54
Ø	9.5 ± 1.1	1.0:1.8 ± 0.4	---	1.52 ± 0.03

## Supplementary Materials

The following are available online at http://www.mdpi.com/2079-4991/8/6/405/s1. Figure S1, Top-view SEM images of CuInS_2_ films synthesized with l-cysteine for different reaction times at 150 °C. Shown are film_9 h, film_15 h and film_21 h. Figure S2, SE image of an exemplary FIB cross section for the film thickness determination of film_140 °C is shown, displaying large fluctuations in the film thickness due to the agglomerates. Figure S3, XRD pattern of CuInS_2_ films synthesized with l-cysteine for different reaction times, 6, 9, 15, 18, 21 and 48 h, at 150 °C. Signals stemming from FTO are marked with *, the ones originating from CuInS_2_ with #. Figure S4, UV-vis spectra of CuInS_2_ thin films on FTO substrate, synthesized solvothermally with l-cysteine grown for different reaction times at 150 °C. Figure S5, (a) HR TEM image and (b) electron diffraction pattern of a CuInS_2_ film synthesized with l-cysteine for 3 h at 150 °C (film_3 h). Figure S6, (a) SEM image and (b) XRD pattern of film_100 °C. Only pure FTO can be observed in SEM and XRD. Figure S7, XRD pattern of CuInS_2_ films synthesized with l-cysteine at different reaction temperatures, 100, 140, 150, and 180 °C, for 18 h. Signals stemming from FTO are marked with *, the ones originating from CuInS_2_ with #. Figure S8, UV-vis spectra of CuInS_2_ thin films on FTO substrate, synthesized solvothermally with l-cysteine grown for 18 h at different reaction temperatures.
