# Supplementary material for: Facile and Robust Solvothermal Synthesis of Nanocrystalline CuInS2 Thin Films
Source: Nanomaterials (Basel). 2018 Jun 5;8(6):405. doi: 10.3390/nano8060405 (PMC6027332; doi:10.3390/nano8060405)
Supplement: Supplementary file 1 [file nanomaterials-08-00405-s001.pdf]

Supporting Information

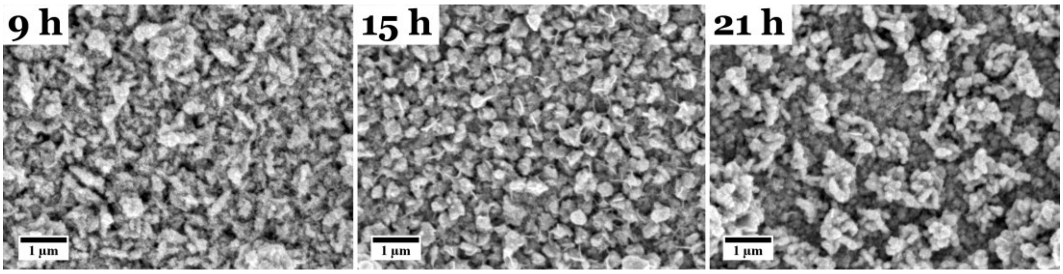

Figure S1. Top-view SEM images of $CuInS_2$ films synthesized with L-cysteine for different reaction times at 150 °C. Shown are film_9h, film_15h and film_21h.

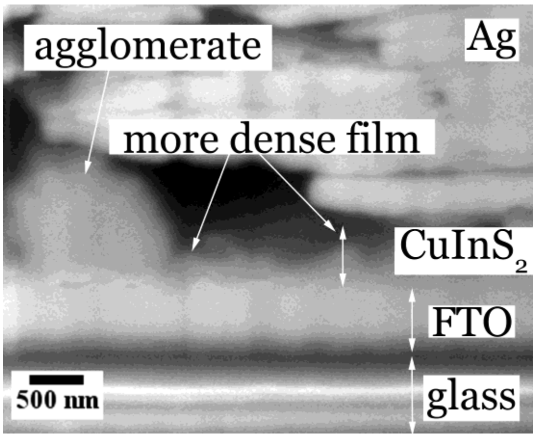

Figure S2. FIB SE image of an exemplary FIB cross section for the film thickness determination of film_140 °C is shown, displaying large fluctuations in the film thickness due to the agglomerates.

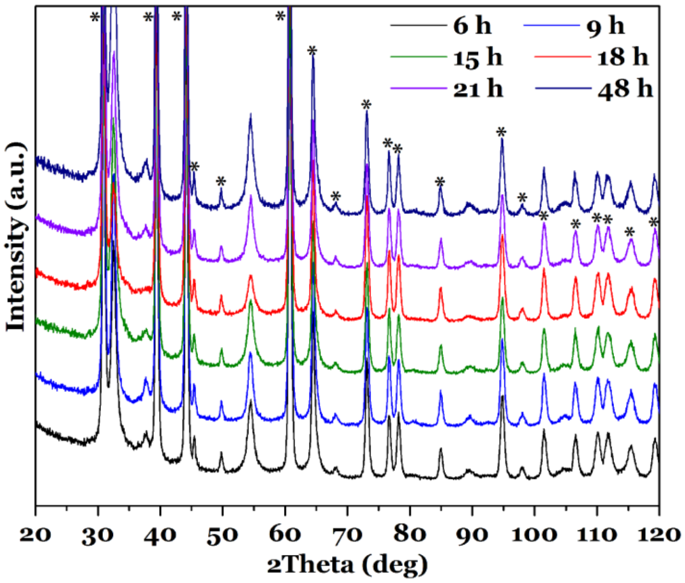

Figure S3. XRD pattern of $CuInS_2$ films synthesized with L-cysteine for different reaction times, 6, 9, 15, 18, 21 and 48 h, at 150 °C. Signals stemming from FTO are marked with *, the ones originating from $CuInS_2$ with #.

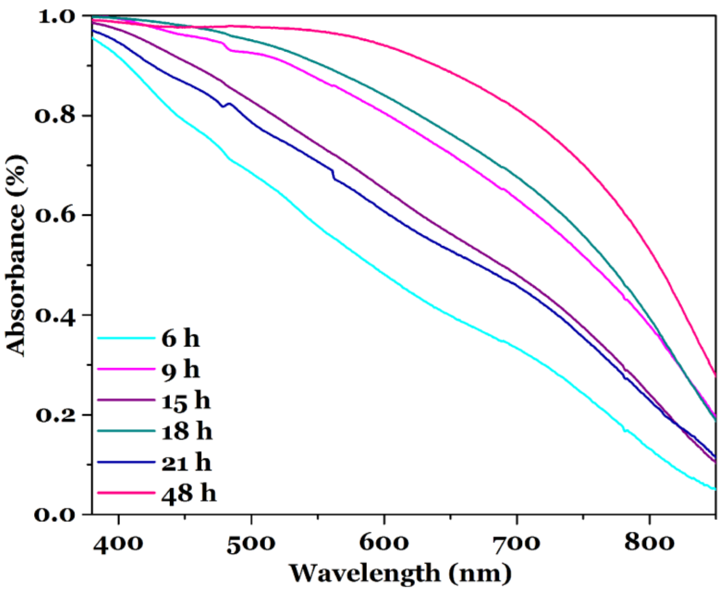

Figure S4. UV-vis spectra of $CuInS_2$ thin films on FTO substrate, synthesized solvothermally with L-cysteine grown for different reaction times at 150 °C.

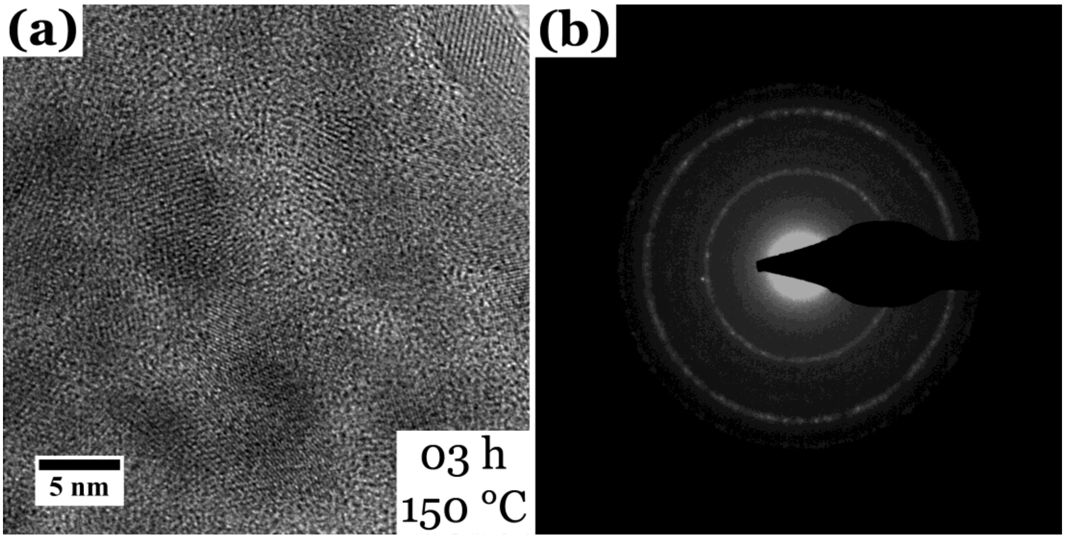

Figure S5. (a) HR TEM image and (b) electron diffraction pattern of a $CuInS_2$ film synthesized with L-cysteine for 3 h at 150 °C (film_3h).

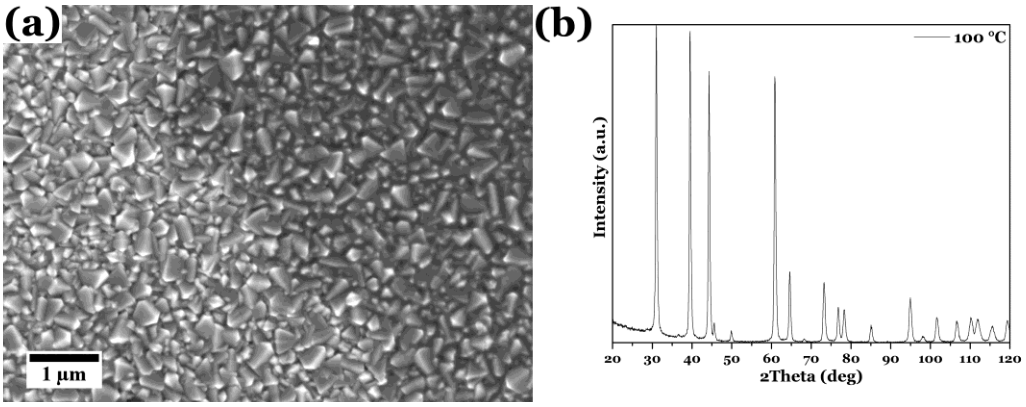

Figure S6. (a) SEM image and (b) XRD pattern of film_100 °C. Only pure FTO can be observed in SEM and XRD.

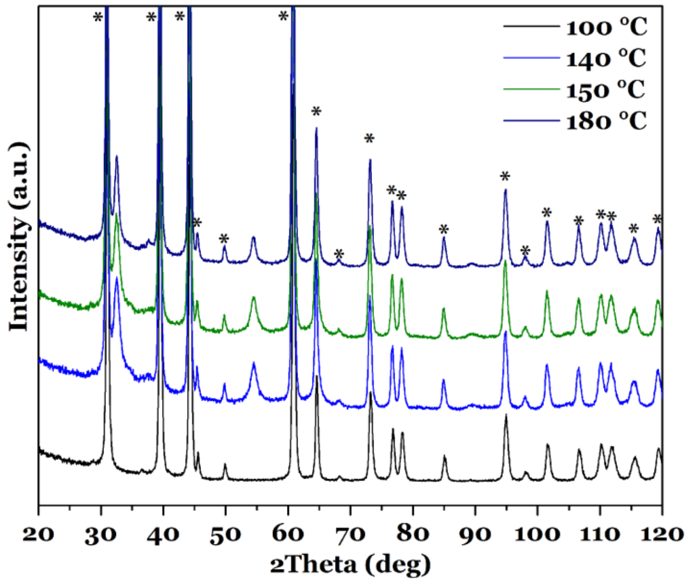

Figure S7. XRD pattern of $CuInS_2$ films synthesized with L-cysteine at different reaction temperatures, 100, 140, 150, and 180 °C, for 18 h. Signals stemming from FTO are marked with *, the ones originating from $CuInS_2$ with #.

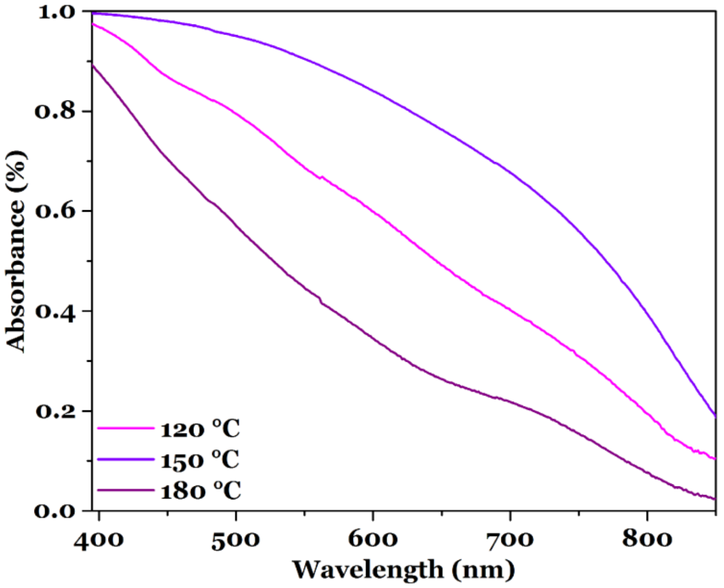

Figure S8. UV-vis spectra of $CuInS_2$ thin films on FTO substrate, synthesized solvothermally with L-cysteine grown for 18 h at different reaction temperatures.
